# Chemokine and Free Fatty Acid Levels in Insulin-Resistant State of Successful Pregnancy: A Preliminary Observation

**DOI:** 10.1155/2012/432575

**Published:** 2012-02-09

**Authors:** Katsuhiko Naruse, Taketoshi Noguchi, Toshiyuki Sado, Taihei Tsunemi, Hiroshi Shigetomi, Seiji Kanayama, Juria Akasaka, Natsuki Koike, Hidekazu Oi, Hiroshi Kobayashi

**Affiliations:** Department of Obstetrics & Gynecology, Nara Medical University, 840, Shijo-cho, Kashihara City, 6348521 Nara, Japan

## Abstract

Increased insulin resistance and inflammatory action are observed in pregnancy-induced hypertension (PIH), but similar insulin resistance is observed also in successful pregnancy. To estimate insulin resistance and inflammatory activity in normal pregnancy and PIH, serum concentrations of free fatty acids (FFA; corrected with albumin to estimate unbound FFA), monocyte chemoattractant protein (MCP)-1, and high-molecular weight (HMW) adiponectin were measured in severe PIH patients with a BMI less than 25 kg/m^2^ and were measured 3 times during the course of pregnancy in women with normal pregnancies. FFA/albumin, MCP-1, and HMW adiponectin concentrations were significantly higher in PIH patients than in women with normal pregnancies. The 3 measurements of FFA/albumin showed a significant increase through the course of uncomplicated pregnancies. In contrast, MCP-1 and HMW adiponectin were significantly decreased during the course of pregnancy. These results suggest that the reduced MCP-1 concentration in normal pregnancy may be a pathway to inhibit the induction of pathological features from physiological insulin resistance and homeostatic inflammation.

## 1. Introduction

Pregnancy-induced hypertension (PIH), a leading complication in pregnancy that affects the mother and fetus, is becoming more frequent mainly because of increasing maternal age [1]. Maternal obesity, a basal condition that increases the risk of PIH 3-fold [2], has also increased over the decades [3]. On the other hand, increased insulin resistance is observed in PIH as well as in successful pregnancies [4–6]. In the course of a normal pregnancy, insulin resistance is correlated with increased maternal adipose tissue deposition [5] and supports placental formation and fetal growth. 

Although the placenta is a large producer of cytokines during pregnancy [7], adipose tissue is regarded as the main organ producing insulin resistance and related cytokines [5, 6, 8, 9]. Recently, free fatty acids (FFA; also known as nonesterified fatty acids (NEFA)) were shown to be mediators of immune and inflammatory actions in adipose tissue [6, 8–13]. Although increased circulating FFA have been observed in gestational diabetes mellitus, preterm delivery, or other adverse maternal outcomes in pregnant subjects [6, 9, 14, 15], increased circulating FFA have also been described in normal pregnancy [16]. Different pathways which do not induce systemic inflammation observed in PIH [4] in insulin resistance remain unclear.

In this study, we measured peripheral monocyte chemoattractant protein-1 (MCP-1), a proinflammatory chemokine that induces monocyte action leading to cell adhesion and endothelial dysfunction, FFA, and high-molecular weight (HMW) adiponectin, a major adipocytokine that reflects insulin sensitivity, in PIH patients and made repeated measurements of these molecules in women with normal pregnancies throughout the course of pregnancy. We hypothesized that an alteration of the chemokines in the inflammatory pathway protects women with normal pregnancies, but not PIH patients, from cardiovascular disorders in a state of physiological insulin resistance.

## 2. Materials and Methods

### 2.1. Subjects

This study was reviewed and approved by the Institutional Review Board of Nara Medical University, and informed consent was obtained from each subject. For the preliminary study, we recruited 17 nonpregnant women with body mass index (BMI) under 25 kg/m^2^, 25 normal pregnant women at 28 weeks or later of gestation, and 7 severe PIH patients. The pregnant women had BMIs under 25 kg/m^2^ prior to pregnancy. All women were East Asian, and none were taking any medications or showed evidence of any metabolic diseases or complications other than PIH. Severe PIH was defined as the new onset of 2 consecutive measurements of diastolic blood pressure ≥110 mmHg and systolic blood pressure ≥160 mmHg diagnosed after 20 weeks of gestation. After the preliminary study, we recruited 36 normal pregnant women for sample correction by taking measurements 3 times throughout the course of pregnancy (1st screening = around 12 weeks of gestation; 2nd = 28 weeks; and 3rd = 36 weeks) for a longitudinal study and paired analysis. All subjects had serum samples available for analysis and did not have gestational diabetes mellitus, thyroid malfunction, or other complications except hypertension. Proteinuria was not considered within the criteria of this study.

All venous blood samples were obtained after an overnight fast at routine medical examination. Serum was separated immediately and stored at −80°C for 3 years for the longest and 6 month for the shortest storage.

### 2.2. Enzyme Immunoassays

Serum FFA (mainly palmitic acid) were measured in duplicate with a commercially available kit (BioVision Research Products, Mountain View, CA). The lower limit of detection was 2 *μ*M. To estimate alterations in unbound FFA, the data were corrected with serum albumin concentrations (FFA (*μ*M)/albumin (g/dL)) by using the BCG albumin assay kit (BioChain, Hayward, CA). The lower limit of detection of albumin was 0.01 g/dL. Serum HMW adiponectin level and its ratio to total adiponectin were measured on the same 96-well plate in duplicate using a commercially available protease-pretreated ELISA kit (Sekisui Medical, Co., Ltd., Japan). The lower limit of detection was 0.075 ng/mL. The intraassay coefficient of variation (CV) was within ±20%, while the inter-assay CV was not more than 15%. Serum leptin and MCP-1 concentrations were measured in duplicate with commercially available ELISA kits (R&D Systems, Inc., Minneapolis, MN). The lower limit of detection was less than 7.8 pg/mL for leptin and less than 5.0 pg/mL for MCP-1. For leptin, the intraassay CV was 3.3% at a concentration of 64.5 pg/mL, 3.0% at 146 pg/mL, and 3.2% at 621 pg/mL, while the inter-assay CV was 5.4% at 65.7 pg/mL, 4.2% at 146 pg/mL, and 3.5% at 581 pg/mL. For MCP-1, the intraassay CV was 7.8% at a concentration of 76.7 pg/mL, 4.7% at 364 pg/mL, and 4.9% at 1121 pg/mL, while the inter-assay CV was 6.7% at 74.2 pg/mL, 5.8% at 352 pg/mL, and 4.6% at 1076 pg/mL. In the longitudinal study, measurement of FFA, albumin, MCP-1, and HMW adiponectin were performed using the techniques described above.

### 2.3. Statistical Analysis for Human Serum Measurement

In the preliminary study, we compared normal pregnant women with nonpregnant women and compared PIH patients with normal pregnant women at 28 weeks or later of gestation. Statistical analysis was performed using the Mann-Whitney *U*-test (SPSS 15.0J; SPSS Japan Inc., Japan). In the longitudinal study, results in respective patients were analyzed in pairs using repeated measures of ANOVA with the post hoc test (Bonferroni correction; SPSS 15.0J). Statistical significance was set at *P* < 0.05. All values are expressed as the mean ±SEM.

## 3. Results

### 3.1. Preliminary Study: FFA and Other Adipocyte-Derived Inflammatory Factors in PIH

In the preliminary study, we compared normal pregnant women at 28 weeks or later of gestation with nonpregnant subjects and compared PIH patients with normal pregnant women at 28 weeks or later of gestation. Subject characteristics are shown in Table 1. Diastolic blood pressure was significantly lower in normal pregnant subjects than in the nonpregnant subjects. Blood pressure values were significantly higher in PIH subjects than in normal pregnant women.

Serum concentrations of FFA (raw data and after correction with albumin), MCP-1, total and HMW adiponectin (raw data and ratio), and leptin are shown in Table 1. FFA concentrations were significantly higher in PIH subjects than in normal pregnant women but no significant difference was observed between normal pregnancy and nonpregnant controls. However, after the albumin correction (an estimated value reflecting unbound FFA), serum concentrations were significantly higher in normal pregnancy than in nonpregnant controls. Serum concentrations of MCP-1 were significantly lower in normal pregnant subjects than in nonpregnant controls and were significantly higher in PIH than in normal pregnant subjects. HMW adiponectin concentration and its ratio to total adiponectin were significantly lower in normal pregnant subjects than in nonpregnant subjects and were higher in PIH subjects than in normal pregnant women. Serum leptin was significantly increased only in PIH patients compared to that in normal pregnant subjects. These trends in HMW adiponectin, leptin, and MCP-1 are similar to those in our former report that included subjects with BMIs greater than 25 kg/m^2^ [17]; however, the trends in total adiponectin and HMW-to-total adiponectin ratio differ from those in our previous report.

### 3.2. Longitudinal Study: FFA, MCP-1, and HMW Adiponectin during the Course of Normal Pregnancy

**Figure 1:**
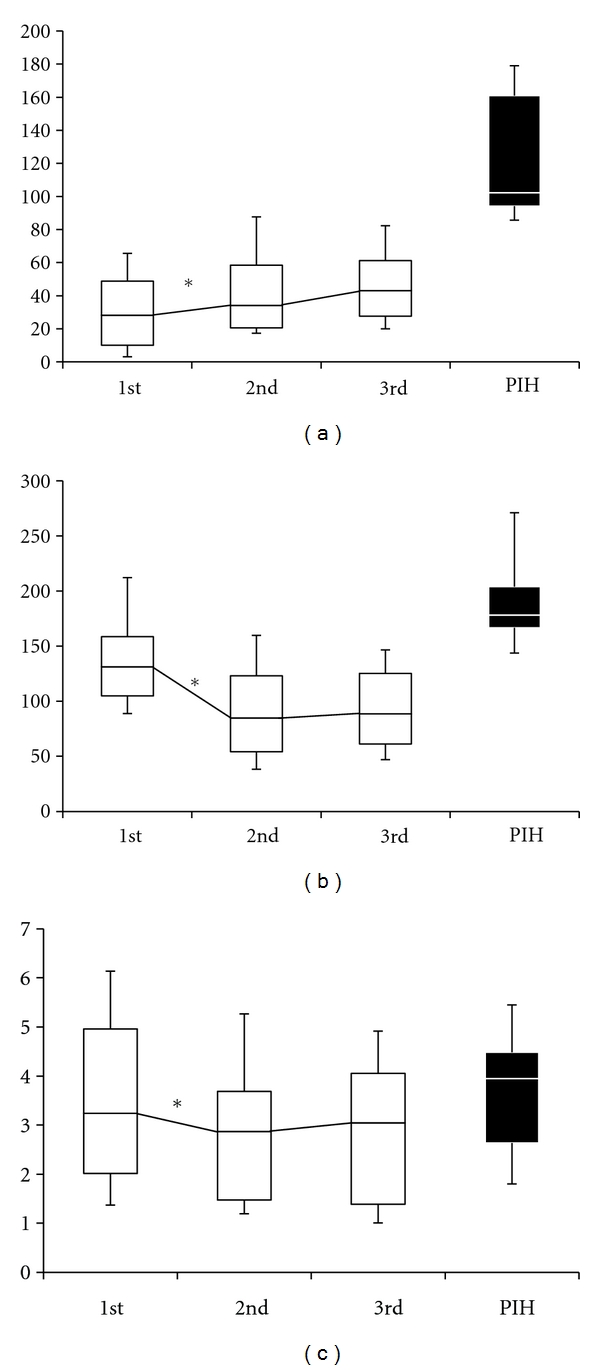
(a) Serum concentrations of FFA (*μ*M)/albumin (g/dL), (b) MCP-1 (pg/mL), and (c) high-molecular weight (HMW) adiponectin (*μ*g/mL) during normal pregnancy and PIH (for reference; see Table 1). In normal pregnancy, samplings were performed 3 times in each subject (see Table 2) and analyzed in pairs. Data are shown as the 90th, 75th, 50th, 25th, and 10th percentile of each measurement group. *P < 0.05 in repeated measures of ANOVA with the post hoc test.

Subject characteristics are shown in Table 2. FFA concentrations were not significantly altered in normal pregnant women over the course of 3 measurements: 1st screening, 97.47 ± 13.05 *μ*M; 2nd, 110.89 ± 12.78; 3rd, 120.85 ± 12.24. However, after the albumin correction (1st screening, 3.15 ± 0.04 g/dL; 2nd, 2.57 ± 0.03; 3rd, 2.52 ± 0.03) to estimate the alteration of unbound FFA, the value (FFA [*μ*M]/albumin [g/dL]) was significantly increased throughout the course of pregnancy: 1st screening, 31.38 ± 4.29; 2nd, 42.51 ± 4.82; 3rd, 48.45 ± 5.10; *P* = 0.0048 (Figure 1). In contrast, MCP-1 concentrations decreased significantly during the course of pregnancy: 1st screening, 154.36 ± 20.27 pg/mL; 2nd, 110.56 ± 33.44; 3rd, 108.78 ± 28.17; *P* < 0.0001 (Figure 1). HMW adiponectin was also significantly decreased during the course of pregnancy: 1st screening, 3.48 ± 0.30 *μ*g/mL; 2nd, 2.88 ± 0.27; 3rd, 2.86 ± 0.25; *P* = 0.0001 (Figure 1).

## 4. Discussion

 Our preliminary study showed increases of FFA (particularly bioactive unbound FFA) and MCP-1 in lean severe PIH patients. However, in normal pregnancy, FFA increased but MCP-1 significantly decreased in the serum. In longitudinal study throughout normal pregnancy, increase of FFA and the decrease of MCP-1 during the course have been clarified. A significant decrease in HMW adiponectin, which may be consistent with the physiological increase of insulin resistance in normal pregnancy, was also confirmed.

 Fatty acids play pivotal roles in the development of several diseases including adult metabolic syndrome [8, 10–12] and pregnancy complications such as miscarriage [6] or preterm delivery [14], though fatty acids are also involved in the successful physiological distribution of energy in pregnancy [18]. It was recently revealed that FFA are mediators of toll-like receptor (TLR)-4 and the NF-kappaB pathway of macrophages within adipose tissue and are regarded as key molecules in systemic inflammation, which plays a role in type 2 diabetes and cardiovascular disease [10, 12, 13]. As we reviewed in this journal [19], TLRs may contribute to pregnancy pathologies. Several reports described increased FFA in preeclampsia [6, 15] but these reports did not estimate unbound FFA. In preeclamptic patients, acute inflammation is one of the major features of preeclampsia pathophysiology [2, 4, 6, 7, 15]. A difference between our study and former studies is that we chose only lean subjects, who may not show adipocyte hypertrophy. Adiponectin was increased in our PIH subjects. We recently reported that increased brain-type natriuretic peptide (BNP) correlated with increased adiponectin in PIH [17], similar to the findings in acute coronary syndrome [20] and cardiomyopathy [21]. We also showed that BNP induced the release of adiponectin from cultured adipocytes *in vitro* [17]. In this respect, further research is needed to reveal the conditions of adipose tissue in PIH patients without obesity.

 MCP-1 is a major chemokine and proinflammatory cytokine that activates monocyte recruitment and strongly contributes systemically to the pathology of inflammation. It is now well accepted that the insulin-resistant state induces the mitogen-activated protein kinase pathway and increases MCP-1 secretion from adipocytes [11, 12], indicating that MCP-1 potentiates the pathology of insulin resistance. Increased MCP-1 was observed in pregnant women with severe obesity [22, 23] and preeclampsia [17, 24]. Additionally, MCP-1 secretion was reported from the human early invasive trophoblast [25]. However, a peripheral decrease of MCP-1 in successful human pregnancy has only been described in a single report using multiple cytokine arrays [26], showing a similar decrease of the serum concentration of this molecule during pregnancy. This decrease may be a system adaptation in humans to avoid pathologic activation of monocytes in pregnancy-induced insulin resistance. The only evidence to support this hypothesis was reported in spontaneously hypertensive rats. MCP-1 expression was increased in the kidney in rat but expression declined significantly after the rats became pregnant, and blood pressure was also decreased [27]. This paper suggests the existence of an adaptation system during pregnancy via chemokine regulation.

 The limitation of this study is that food intake was not equalized between each sampling even though all samples were taken after an overnight fast. FFA concentrations are altered for several days after different food choices, and albumin may decrease with emesis or anemia. A larger cohort study or more frequent sampling may reduce the alteration of the results after an unusual dietary event.

## 5. Conclusions

 Although FFA were also increased during the course of normal pregnancy, which may be consistent with physiological insulin resistance, MCP-1 was decreased, which would inhibit MCP-1-mediated pathologic inflammation during the hyperlipidemic state of a successful pregnancy. FFA and MCP-1 in adipose tissue are regarded as key molecules in homeostatic inflammatory linkage. This is the first report suggesting a difference between pathological inflammation and reasonable insulin resistance in human pregnancy. Further research in adipocytokines and adipose tissue may lead new statistics for prediction and therapy of pregnancy complications.

## Figures and Tables

**Table 1:** Characteristic of the subjects and serum concentrations of the molecules.

	Nonpregnant control	Normal pregnancy later than 28 weeks	Pregnancy-induced hypertension
*n*	17	25	7
Gestational age at sampling (weeks)		33.2 ± 0.7	32.3 ± 0.8
BMI at sampling (kg/m^2^)	20.3 ± 0.4	23.9 ± 0.5*	24.5 ± 0.6
BMI before pregnancy (kg/m^2^)	20.3 ± 0.4	20.5 ± 0.4	20.9 ± 0.9
Blood pressure			
Systolic	106.3 ± 1.8	107.3 ± 1.8	174.8 ± 2.0
Diastolic	73.1 ± 1.2	58.8 ± 1.6*	100.3 ± 2.6**
MAP	84.1 ± 1.1	74.9 ± 1.5*	107.3 ± 17.9**

FFA (*μ*M)	155.62 ± 29.17	153.68 ± 16.23	236.54 ± 31.89**
Albumin (g/dL)	3.48 ± 0.07	2.62 ± 0.05*	1.92 ± 0.12**
FFA/Alb	41.62 ± 8.50	58.82 ± 5.94*	124.94 ± 15.96**
MCP-1 (pg/mL)	219.50 ± 12.17	131.95 ± 8.63*	195.41 ± 27.22**
Adiponectin			
Total (*μ*g/mL)	8.85 ± 0.62	5.33 ± 0.37*	6.82 ± 1.00
HMW (*μ*g/mL)	4.45 ± 0.46	1.97 ± 0.20*	3.66 ± 0.62**
HMW/total adiponectin ratio	0.49 ± 0.03	0.36 ± 0.02*	0.53 ± 0.04**
Leptin (ng/mL)	6.91 ± 0.91	8.31 ± 1.09	35.56 ± 10.31**

BMI: body mass index; MAP: mean arterial pressure; FFA: free fatty acids; MCP: monocyte chemotactic protein; HMW: high-molecular weight.

*P < 0.05 versus nonpregnant control.

**P < 0.05 versus normal pregnancy later than 28 weeks.

**Table 2:** Characteristic of the subjects of longitudinal study.

*n*	36
Maternal age at delivery (years)	30.9 ± 0.7
Parity (times)	
0	18
1	13
2 or more	5
Average gestational age at sampling (weeks^+days^)	
1st	11^+5^ (8^+2^–14^+4^)
2nd	28^+3^ (27^+2^–29^+5^)
3rd	36^+1^ (35^+0^–37^+2^)
BMI before pregnancy (kg/m^2^)	22.1 ± 0.6
BMI on delivery (kg/m^2^)	26.4 ± 0.6
Average gestational age at delivery (weeks^+days^)	39^+5^ (36^+6^–41^+3^)
Infant birth weight (g)	3052.0 ± 78.7

BIM: body mass index.

## Abbreviations

PIH: Pregnancy-induced hypertension
FFA: Free fatty acids
NEFA: Nonesterified fatty acids
MCP-1: Monocyte chemoattractant protein-1
HMW: High molecular weight
BMI: Body mass index.

## References

[1] Matsuda Y, Kawamichi Y, Hayashi K (2011). Impact of maternal age on the incidence of obstetrical complications in Japan. *Journal of Obstetrics and Gynaecology Research*.

[2] Roberts JM, Bodnar LM, Patrick TE, Powers RW (2011). The role of obesity in preeclampsia. *Pregnancy Hypertension*.

[3] Heslehurst N, Ells LJ, Simpson H, Batterham A, Wilkinson J, Summerbell CD (2007). Trends in maternal obesity incidence rates, demographic predictors, and health inequalities in 36 821 women over a 15-year period. *BJOG*.

[4] Seely EW, Solomon CG (2003). Insulin resistance and its potential role in pregnancy-induced hypertension. *Journal of Clinical Endocrinology and Metabolism*.

[5] Valsamakis G, Kumar S, Creatsas G, Mastorakos G (2010). The effects of adipose tissue and adipocytokines in human pregnancy. *Annals of the New York Academy of Sciences*.

[6] Jarvie E, Hauguel-de-Mouzon S, Nelson SM, Sattar N, Catalano PM, Freeman DJ (2010). Lipotoxicity in obese pregnancy and its potential role in adverse pregnancy outcome and obesity in the offspring. *Clinical Science*.

[7] Roberts KA, Riley SC, Reynolds RM (2011). Placental structure and inflammation in pregnancies associated with obesity. *Placenta*.

[8] Jensen MD (2008). Role of body fat distribution and the metabolic complications of obesity. *Journal of Clinical Endocrinology and Metabolism*.

[9] Schaefer-Graf UM, Graf K, Kulbacka I (2008). Maternal lipids as strong determinants of fetal environment and growth in pregnancies with gestational diabetes mellitus. *Diabetes Care*.

[10] Schaeffler A, Gross P, Buettner R (2009). Fatty acid-induced induction of Toll-like receptor-4/nuclear factor-*κ*B pathway in adipocytes links nutritional signalling with innate immunity. *Immunology*.

[11] Ito A, Suganami T, Miyamoto Y (2007). Role of MAPK phosphatase-1 in the induction of monocyte chemoattractant protein-1 during the course of adipocyte hypertrophy. *Journal of Biological Chemistry*.

[12] Suganami T, Ogawa Y (2010). Adipose tissue macrophages: their role in adipose tissue remodeling. *Journal of Leukocyte Biology*.

[13] Lee JY, Sohn KH, Rhee SH, Hwang D (2001). Saturated fatty acids, but not unsaturated fatty acids, induce the expression of cyclooxygenase-2 mediated through Toll-like receptor 4. *Journal of Biological Chemistry*.

[14] Chen X, Scholl TO (2008). Association of elevated free fatty acids during late pregnancy with preterm delivery. *Obstetrics and Gynecology*.

[15] Villa PM, Laivuori H, Kajantie E, Kaaja R (2009). Free fatty acid profiles in preeclampsia. *Prostaglandins Leukotrienes and Essential Fatty Acids*.

[16] Sivan E, Boden G (2003). Free fatty acids, insulin resistance, and pregnancy. *Current Diabetes Reports*.

[17] Naruse K, Yamasaki Y, Tsunemi T (2011). Increase of high molecular weight adiponectin in hypertensive pregnancy was correlated with brain-type natriuretic peptide stimulation on adipocyte. *Pregnancy Hypertension*.

[18] Herrera E, Amusquivar E (2000). Lipid metabolism in the fetus and the newborn. *Diabetes/Metabolism Research and Reviews*.

[19] Noguchi T, Sado T, Naruse K (2010). Evidence for activation of toll-like receptor and receptor for advanced glycation end products in preterm birth. *Mediators of Inflammation*.

[20] Ang DSC, Welsh P, Watt P, Nelson SM, Struthers A, Sattar N (2009). Serial changes in adiponectin and BNP in ACS patients: paradoxical associations with each other and with prognosis. *Clinical Science*.

[21] Kitaoka H, Kubo T, Okawa M (2010). Plasma adiponectin levels and left ventricular remodeling in hypertrophic cardiomyopathy. *International Heart Journal*.

[22] Madan JC, Davis JM, Craig WY (2009). Maternal obesity and markers of inflammation in pregnancy. *Cytokine*.

[23] Basu S, Haghiac M, Surace P (2011). Pregravid obesity associates with increased maternal endotoxemia and metabolic inflammation. *Obesity*.

[24] Lockwood CJ, Matta P, Krikun G (2006). Regulation of monocyte chemoattractant protein-1 expression by tumor necrosis factor-*α* and interleukin-1*β* in first trimester human decidual cells: implications for preeclampsia. *American Journal of Pathology*.

[25] Naruse K, Innes BA, Bulmer JN, Robson SC, Searle RF, Lash GE (2010). Secretion of cytokines by villous cytotrophoblast and extravillous trophoblast in the first trimester of human pregnancy. *Journal of Reproductive Immunology*.

[26] Kraus TA, Sperling RS, Engel SM (2010). Peripheral blood cytokine profiling during pregnancy and post-partum periods. *American Journal of Reproductive Immunology*.

[27] Iacono A, Bianco G, Mattace Raso G (2009). Maternal adaptation in pregnant hypertensive rats: improvement of vascular and inflammatory variables and oxidative damage in the Kidney. *American Journal of Hypertension*.

